# Condylar Positional Changes Following Manual Proximal Segment Positioning During Bilateral Sagittal Split Ramus Osteotomy: A Cephalometric Study

**DOI:** 10.3390/medicina62061154

**Published:** 2026-06-13

**Authors:** Nuri Can Tanrısever, Hatice Gökalp

**Affiliations:** Department of Orthodontics, School of Dentistry, University of Ankara, Ankara 06560, Türkiye

**Keywords:** cephalometry, mandibular condyle, orthognathic surgery, osteotomy, sagittal split ramus, temporomandibular joint

## Abstract

*Background and Objectives*: Maintenance of condylar position during bilateral sagittal split ramus osteotomy (BSSRO) is important for preserving temporomandibular joint biomechanics and skeletal stability. During surgery, loss of muscle tone under general anesthesia may alter the condyle–fossa relationship, making accurate repositioning of the proximal segment challenging. Although manual positioning remains the most commonly used intraoperative approach, evidence regarding its ability to preserve the preoperative condyle–fossa relationship remains limited. This study evaluated changes in the condyle–fossa relationship following BSSRO performed with manual proximal segment positioning. *Materials and Methods*: This single-center retrospective study included lateral cephalometric radiographs of 14 patients (8 females, 6 males; aged 19–29 years) with skeletal Class III malocclusion treated with combined orthodontic treatment and BSSRO. Radiographs were obtained preoperatively (T0), immediately postoperatively (T1), and at the final follow-up examination (T2). Condylar position was assessed using a Cartesian coordinate system, joint space measurements, and the Condyle Position Index (CPI). Statistical analyses were performed using the Friedman and Wilcoxon signed-rank tests (*p* < 0.05). *Results*: Significant differences were observed in CPI and anterior joint space measurements across the observation periods. Interval analysis demonstrated increased CPI values and decreased anterior joint space measurements between T1 and T2, whereas no significant immediate postoperative changes were observed. Intra-observer reliability was excellent, with intraclass correlation coefficients exceeding 0.90 for all variables. *Conclusions*: Manual positioning of the proximal segment during BSSRO may provide acceptable immediate postoperative condyle–fossa stability but may not completely maintain the preoperative condyle–fossa relationship over time. Although no significant immediate postoperative changes were observed, significant changes in the condyle–fossa relationship were identified at the final follow-up examination. These findings support the need for further prospective studies incorporating clinical temporomandibular joint assessment and three-dimensional imaging.

## 1. Introduction

Stable mandibular position is primarily maintained by the coordinated activity of the masticatory muscles rather than occlusal contact. Physiologically, the mandible is considered most stable when the condyles are seated in a supero-anterior position within the glenoid fossae, stabilized by the elevator muscles and the lateral pterygoid muscle, with the articular discs properly interposed. This musculoskeletally stable position (MSSP) is widely accepted as the clinical definition of centric relation and serves as the physiologic reference position for diagnosis and treatment planning in dentistry [1,2].

Preservation of this relationship between the temporomandibular joint (TMJ) components and occlusion is fundamental not only to dental treatment, but also to craniofacial functional stability, joint biomechanics, and long-term postoperative adaptation following surgical correction of dentofacial deformities. Orthognathic surgery is an established treatment modality for correcting skeletal discrepancies affecting facial esthetics, occlusal function, and masticatory efficiency [3]. Among these procedures, mandibular osteotomies play a critical role in the management of skeletal Class III deformities and other mandibular positional abnormalities.

In mandibular orthognathic procedures, particular attention must be given to the positional stability of the proximal segment, which carries the condyle, as alterations in condylar position may compromise TMJ biomechanics and contribute to postoperative joint dysfunction, skeletal relapse, and adaptive remodeling of the joint structures [4,5].

During bilateral sagittal split ramus osteotomy (BSSRO), the loss of muscle tone under general anesthesia allows the proximal segment to move freely, predisposing the condyle to positional alterations. Previous studies have reported that condylar displacement occurs in a substantial proportion of mandibular prognathism surgeries, frequently resulting in posterior positioning of the condyle within the glenoid fossa [6,7,8,9].

To minimize this risk, several intraoperative strategies have been proposed, including rigid fixation techniques, navigation systems, sonographic guidance, and manual positioning of the proximal segment. Although these approaches aim to improve condylar repositioning, accurate reproduction of the preoperative condyle–fossa relationship remains challenging. In particular, the accuracy of manual positioning is highly dependent on the surgeon’s experience and intraoperative judgment. It has been reported that counterclockwise rotation of the proximal segment during manual positioning may lead to posterior displacement of nearly half of the condyles, although some may return toward their initial position during the postoperative period [10,11,12].

Although various condylar positioning techniques have been described, manual proximal segment positioning remains the most frequently used approach in routine orthognathic practice because of its practicality and low intraoperative burden. However, objective evidence regarding its ability to preserve the physiologic condylar relationship, particularly in relation to postoperative TMJ stability and skeletal outcomes, remains limited. In addition, most previous investigations have primarily focused on immediate postoperative condylar displacement, whereas changes in condylar position during the subsequent follow-up period have been less frequently investigated under standardized cephalometric conditions.

Therefore, the aim of this study was to cephalometrically evaluate changes in condylar position before and after bilateral sagittal split ramus osteotomy performed using manual positioning of the proximal segment. The null hypothesis was that manual intraoperative control of the proximal segment does not ensure reproducible maintenance of the preoperative condylar position.

## 2. Materials and Methods

### 2.1. Study Design

This single-center retrospective study was conducted using lateral cephalometric radiographs retrieved from the archive of Ankara University Faculty of Dentistry, Department of Orthodontics. The study sample consisted of 14 patients (8 females, 6 males; age range: 19–29 years) diagnosed with skeletal Class III malocclusion and treated with combined orthodontic treatment and bilateral sagittal split ramus osteotomy. Initially, patients who underwent BSSRO were screened retrospectively from the institutional archive. After application of the inclusion and exclusion criteria, 14 patients with complete radiographic records were included in the final analysis.

Lateral cephalometric radiographs were obtained under standardized conditions at three time points: preoperatively (T0), immediately postoperatively (T1), and at the completion of orthodontic treatment (T2). The T2 radiographs were obtained following completion of postoperative orthodontic treatment, with a mean follow-up period of 7.1 ± 0.8 months.

Within the retrospective study design, standardized lateral cephalometric radiographs were available for all patients at all observation periods (T0, T1, and T2), allowing longitudinal assessment under consistent imaging conditions. Although cone-beam computed tomography (CBCT) provides three-dimensional visualization of condylar morphology and position, lateral cephalometric radiographs have been widely used for the evaluation of sagittal condylar position and condyle–fossa relationships in longitudinal orthodontic and orthognathic studies. Nevertheless, this method is inherently limited to two-dimensional assessment and does not permit direct evaluation of mediolateral condylar displacement or rotational changes.

All surgical procedures were performed under general anesthesia by the same oral and maxillofacial surgeon using a standardized BSSRO protocol. During BSSRO, after mandibular repositioning, the proximal segment was manually seated by the surgeon to reproduce the preoperative condylar relationship within the glenoid fossa before definitive fixation. Fixation of the proximal and distal segments was achieved using bicortical screws. To maintain postoperative occlusion, intermaxillary fixation was applied for one week, followed by intermaxillary elastics. Orthodontic treatment was resumed 30 days after surgery and completed within 6 to 8 months.

The magnitude of mandibular setback achieved during BSSRO was determined using NemoFAB software (version 2020, NemoFAB, Nemotec, Madrid, Spain). Surgical movement was calculated as the horizontal displacement of Point B between the preoperative virtual surgical plan and the postoperative skeletal position. The mean mandibular setback amount was 5.6 ± 1.8 mm (range: 3.0–8.5 mm).

This retrospective study was approved by the Ethics Committee of Ankara University Faculty of Dentistry (Meeting No: 3; Decision Date: 25 November 2024). Due to the retrospective design and use of archived data, informed consent was waived by the ethics committee. All procedures were conducted in accordance with the ethical principles of the Declaration of Helsinki (1975), as revised in 2013.

### 2.2. Inclusion and Exclusion Criteria

Patients were included if they were diagnosed with skeletal Class III malocclusion, underwent BSSRO performed using standardized manual positioning of the proximal segment, had complete lateral cephalometric records at T0, T1, and T2, and had no history of temporomandibular joint disorders or trauma. The absence of temporomandibular disorders was determined retrospectively from the available clinical records. Patients with documented TMJ pain, joint sounds, mandibular movement limitations, previous TMJ treatment, or a history of TMJ trauma were excluded from the study. Patients were excluded if they had craniofacial syndromes or systemic conditions affecting bone metabolism, underwent additional maxillofacial surgical procedures, or had poor-quality or distorted radiographic records. In addition, patients lacking immediate postoperative (T1) or end-of-treatment (T2) lateral cephalometric radiographs were excluded because longitudinal assessment required complete records at all observation periods. A total of 29 patient records were initially screened, and after application of the inclusion and exclusion criteria, 14 patients were eligible and included in the final analysis.

### 2.3. Radiographic Standardization and Calibration

All lateral cephalometric radiographs were obtained using the same cephalostat with standardized head positioning and exposure parameters. During image acquisition, the head was stabilized using the cephalostat positioning system(Carestream Dental 8100, Carestream Health Inc., Rochester, NY, USA) to ensure reproducible orientation and minimize positional variability between radiographs. Therefore, all radiographs were considered to represent standardized cephalometric head positioning for subsequent analysis. To ensure measurement accuracy, the 10 mm reference scale visible on the cephalostat rod was calibrated to 1 cm on each radiograph prior to analysis.

All measurements were performed digitally by the same investigator (N.T.) using AutoCAD software (AutoCAD 2025, Autodesk Inc., San Francisco, CA, USA).

Due to the inherent superimposition of bilateral anatomical structures in lateral cephalometric radiographs, special attention was paid to landmark identification. Landmarks were determined using high-magnification digital zoom and contrast adjustment within the software environment to improve visualization. Only clearly distinguishable cortical outlines were used for point identification. In cases where superimposition affected landmark clarity, the midpoint of overlapping contours was consistently selected according to a standardized protocol. Specifically, when bilateral cortical outlines could not be clearly distinguished, the geometric midpoint between the superimposed contours was identified and recorded as the landmark location. The same approach was applied to all radiographs to ensure consistency throughout the study. Potential landmark identification errors associated with anatomical superimposition were further controlled through intra-observer reliability assessment. The reproducibility of landmark identification and measurements was subsequently evaluated using intraclass correlation coefficients (ICCs), as described in Section 2.6.

### 2.4. Coordinate System and Condylar Reference Points

**Figure 1:**
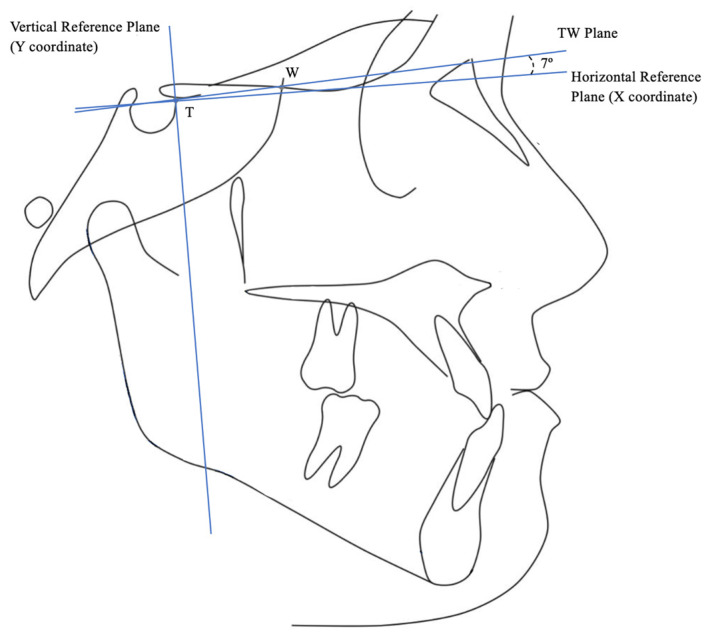
Cartesian coordinate system used for cephalometric assessment. The *X*-axis was established 7° below the TW plane, and the *Y*-axis was drawn perpendicular through point T.

A Cartesian coordinate system was constructed for each radiograph. The *X*-axis (horizontal reference plane) was defined as a plane located 7° below the TW plane formed between the Tuberculum Sella (T) and Wings (W) points, which has been reported as a stable cranial reference for longitudinal cephalometric assessment [13], with point T serving as the origin. The *Y*-axis (vertical reference plane) was drawn perpendicular to the *X*-axis through point T (Figure 1).

Three reproducible reference points were identified on the cortical outline of the condyle: the anterior condylar point (Ca), posterior condylar point (Cp), and superior condylar point (Cs). The linear distances of these points to both the X- and Y-axes were measured in millimeters.

### 2.5. Assessment of Condylar Position in the Glenoid Fossa

**Figure 2:**
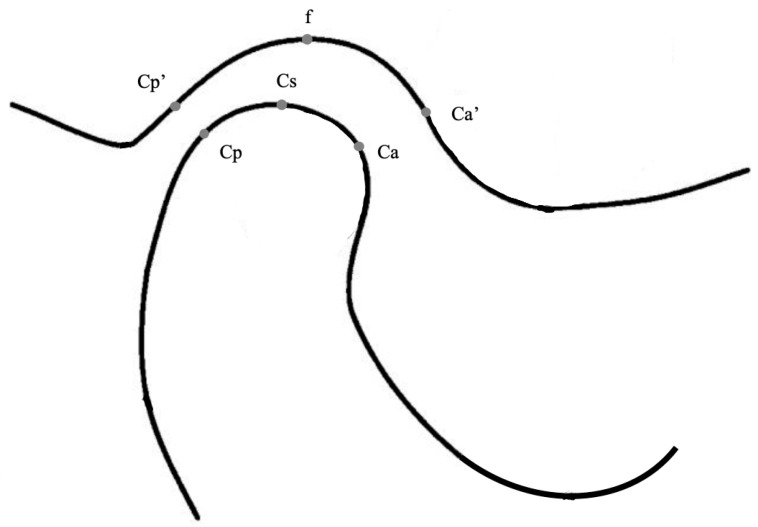
Reference landmarks for TMJ measurements on lateral cephalometric radiographs: f, deepest point of the glenoid fossa; Cs, most superior point of the condyle; Ca and Cp, tangential anterior and posterior condylar points aligned with f; Ca′ and Cp′, projection points on the anterior and posterior walls of the glenoid fossa from perpendicular lines passing through Ca and Cp.

**Figure 3:**
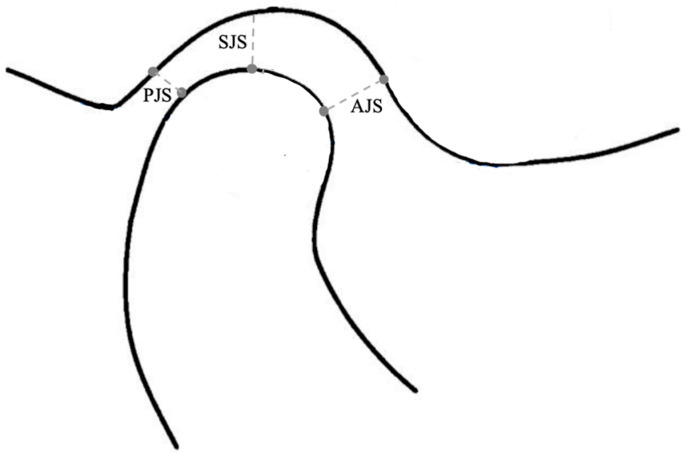
Linear measurements used to assess condylar position: AJS, distance between Ca and Ca′; SJS, distance between Cs and f; PJS, distance between Cp and Cp′.

Anterior joint space (AJS), posterior joint space (PJS), and superior joint space (SJS) were measured on lateral cephalometric radiographs at T0, T1, and T2 to evaluate condylar position within the glenoid fossa (Figure 2 and Figure 3).

Condylar position was quantified using the Condyle Position Index (CPI), based on standardized condylar and joint space measurements, as previously described in the literature [14,15]:

CPI = [(PJS − AJS)/(PJS + AJS)] × 100

Positive CPI values indicated anterior positioning of the condyle, whereas negative values indicated posterior positioning.

### 2.6. Measurement Reliability

To assess intra-observer reliability, 10 randomly selected lateral cephalometric radiographs were re-measured by the same investigator two weeks after the initial measurements under identical conditions.

### 2.7. Statistical Analysis

Statistical analyses were performed using IBM SPSS Statistics software (Version 26, IBM Corp., Armonk, NY, USA). Normality of data distribution was evaluated using the Shapiro–Wilk test. As the measurements did not follow a normal distribution, non-parametric tests were used. Descriptive statistics were presented as median and interquartile range (IQR).

Differences among T0, T1, and T2 measurements were evaluated using the Friedman test. Pairwise comparisons between T0–T1 and T1–T2 were performed using the Wilcoxon signed-rank test. Effect size (r) was calculated as r = Z/√N. Statistical significance was set at *p* < 0.05.

Intra-observer reliability was assessed using the intraclass correlation coefficients (ICCs; two-way mixed-effects model, absolute agreement).

An a priori power analysis was performed using G*Power software (Version 3.1, Heinrich Heine University, Düsseldorf, Germany). Based on a paired-measures design, a large effect size (0.80) according to Cohen’s classification, α = 0.05, and statistical power of 0.80, the minimum required sample size was calculated as 12 participants. Therefore, inclusion of 14 patients was considered sufficient to achieve the intended statistical power.

## 3. Results

Review of the clinical records revealed that none of the patients experienced postoperative infection, bone instability, or malunion following bilateral sagittal split ramus osteotomy.

Lateral cephalometric measurements were used to document immediate postoperative skeletal and occlusal changes following surgery (Table 1).

The mean mandibular setback achieved during BSSRO was 5.6 ± 1.8 mm, with a range of 3.0 to 8.5 mm.

Comparison of condylar and joint space measurements across T0, T1, and T2 demonstrated statistically significant differences for the Condyle Position Index (CPI) and anterior joint space (AJS) (*p* < 0.05; Table 2). Mean CPI values increased from 1.07 ± 13.80 at T0 to 1.54 ± 13.89 at T1 and 7.02 ± 11.12 at T2, indicating a progressive tendency toward a more anterior condyle–fossa relationship over time. Similarly, mean AJS values remained relatively stable between T0 (1.69 ± 0.51 mm) and T1 (1.71 ± 0.56 mm), but decreased to 1.57 ± 0.46 mm at T2. No significant differences were observed for SJS, PJS, or any of the Cartesian coordinate measurements.

Pairwise comparisons revealed that the greatest changes occurred during the T2–T1 interval. Mean CPI increased by 5.49 ± 6.11 during the orthodontic treatment period, compared with only 0.47 ± 4.28 during the immediate postoperative period. Likewise, AJS showed a reduction of 0.14 ± 0.17 mm during the T2–T1 interval, whereas virtually no change was observed during the T1–T0 interval (0.02 ± 0.14 mm). No significant interval-dependent differences were detected for the remaining measurements (Table 3).

Intra-observer reliability analysis demonstrated excellent agreement between repeated measurements, with intraclass correlation coefficients (ICCs) exceeding 0.90 for all variables.

**Table 3:** Pairwise changes in condylar and joint space measurements during the T0–T1 and T1–T2 intervals.

Measurements	T1–T0	T2–T1	*p*
	Mean ± SD	Mean ± SD	
CPI	0.47 ± 4.28	5.49 ± 6.11	0.023
AJS (mm)	0.02 ± 0.14	−0.14 ± 0.17	0.042
SJS (mm)	0.01 ± 0.16	−0.01 ± 0.19	0.856
PJS (mm)	0.03 ± 0.17	0.05 ± 0.15	0.750
Ca-Y (mm)	0.06 ± 1.50	0.02 ± 1.64	0.432
Cs-Y (mm)	0.27 ± 1.68	0.03 ± 2.14	0.624
Cp-Y (mm)	0.04 ± 1.69	0.10 ± 2.33	0.615
Ca-X (mm)	0.79 ± 1.82	−0.18 ± 2.01	0.286
Cs-X (mm)	0.89 ± 2.01	−0.44 ± 1.66	0.140
Cp-X (mm)	0.78 ± 2.06	−0.33 ± 2.06	0.172

Values represent the mean difference ± SD between the indicated time periods. CPI: Condyle Position Index; AJS: anterior joint space; SJS: superior joint space; PJS: posterior joint space. X and Y represent horizontal and vertical distances according to the constructed Cartesian coordinate system. Positive and negative values indicate the direction of change between measurements. *p* < 0.05.

## 4. Discussion

The present study suggests that manual positioning of the proximal segment during BSSRO may not completely maintain the preoperative condylar position throughout the postoperative period. While no significant change was observed between the preoperative and immediate postoperative periods, significant changes in condylar position within the glenoid fossa were observed at the end of treatment, as reflected by increased CPI values and reduced anterior joint space measurements. Because none of the Cartesian coordinate measurements demonstrated significant differences, these findings should be interpreted as changes in the condyle–fossa relationship rather than direct evidence of true condylar displacement. These findings indicate that the condyle–fossa relationship may not have been fully maintained throughout the postoperative period and may have undergone gradual adaptation during healing and orthodontic finishing.

Condylar position has been evaluated using lateral cephalometric radiographs in several orthodontic and orthognathic studies. In the present study, condylar measurements were performed according to the Cartesian coordinate system [13,16,17,18], based on the TW plane, which has been demonstrated to be stable within the middle cranial base [19,20].

The concept of centric relation has been described as an antero-superior condylar position established by the coordinated activity of the masticatory musculature [21,22,23]. Loss of muscle tone under general anesthesia during mandibular ramus surgery allows the condyle to change position under the influence of gravity [6,8,9,12,24]. Therefore, one of the most challenging steps in BSSRO is the accurate repositioning of the condyle within the glenoid fossa [25]. Hollander and Ridell reported that condylar position changed in 70% of Class III mandibular surgeries [7].

Landes and Sterz suggested that posterior positioning of the condyle may result from compression, torque, and rotational forces occurring during ramus surgery [26,27]. In contrast, Spitzer et al. argued that there is no direct relationship between the magnitude of mandibular movement and postoperative condylar position [6,7,8,27,28,29,30,31]. The present findings suggest that, despite intraoperative manual positioning, the condyle–fossa relationship may not remain completely unchanged throughout the postoperative period.

Two possible explanations may account for this observation. First, manual positioning may fail to reproduce the preoperative condyle–fossa relationship precisely. Second, even if an acceptable relationship is achieved intraoperatively, subtle changes may occur during fixation and the postoperative healing period. This interpretation is supported by the temporal pattern observed in the present study, where no significant changes were detected immediately after surgery, whereas significant alterations were observed at the final follow-up examination. The absence of significant differences at T1 suggests that manual positioning may have provided an acceptable immediate postoperative condyle–fossa relationship. However, this relationship may not have remained completely stable throughout the postoperative period. Progressive healing and postoperative remodeling processes may have contributed to gradual alterations in the condyle–fossa relationship that became apparent only at T2. Therefore, the observed findings may reflect both postoperative adaptation and the limited long-term reproducibility of manual proximal segment positioning.

Although there is no clear consensus regarding the relationship between condylar position and temporomandibular joint symptoms [15,32,33,34,35,36], it has been reported that 16.5% of initially asymptomatic patients may develop symptoms postoperatively if the condyle is not correctly positioned during surgery [37,38,39]. In the present study, condylar position was evaluated using the CPI method [13]. A slight tendency toward a more posterior condyle–fossa relationship was observed immediately after surgery, whereas a more anterior condyle–fossa relationship was observed at the end of orthodontic treatment. Interestingly, the increase in CPI values was primarily associated with a reduction in anterior joint space, whereas posterior and superior joint space measurements remained relatively stable throughout the observation period. This pattern may indicate subtle changes in the condyle–fossa relationship within the sagittal plane rather than substantial displacement of the condyle within the glenoid fossa. The absence of significant changes in PJS and SJS suggests that the observed adaptation was localized predominantly to the anterior aspect of the joint space. Furthermore, because none of the Cartesian coordinate measurements demonstrated significant differences, these findings are more appropriately interpreted as changes in the condyle–fossa relationship rather than direct evidence of true condylar displacement. Previous studies have suggested that postoperative changes in condylar position may be influenced by neuromuscular adaptation and alterations in masticatory muscle function following orthognathic surgery [40]. The gradual changes observed in the present study suggest that the condyle–fossa relationship may continue to evolve during the postoperative follow-up period rather than remaining completely unchanged after fixation.

However, the absolute magnitude of the observed changes was relatively small. Although statistically significant differences were detected in CPI and AJS, no universally accepted threshold exists for defining clinically meaningful postoperative changes in these parameters. Furthermore, because TMJ symptoms, functional outcomes, and skeletal stability were not evaluated in the present study, the clinical implications of these radiographic findings should be interpreted with caution. Therefore, the observed differences are more appropriately regarded as subtle radiographic changes in the condyle–fossa relationship rather than direct evidence of postoperative TMJ dysfunction or skeletal instability.

Previous short-term studies evaluating the effect of Class III mandibular surgery on condylar position have reported inconsistent findings [41,42,43,44,45,46,47]. Three-dimensional investigations have demonstrated that measurable condylar positional changes may occur immediately after surgery, although the magnitude and direction of these changes have not been consistent across studies [41,42,43,44,45]. Likewise, Kim et al. reported that condylar position may be influenced by the surgical procedure and may change during the postoperative follow-up period [46,47]. In the present study, no significant immediate postoperative changes were detected, which may suggest that manual proximal segment positioning provided an acceptable short-term condyle–fossa relationship. However, the increase in CPI values and reduction in AJS observed at T2 indicate that the condyle–fossa relationship was not entirely static and may have undergone gradual postoperative adaptation during healing and orthodontic finishing. These findings are generally consistent with previous reports suggesting that condylar position following orthognathic surgery may evolve over time rather than remain unchanged after fixation [42,43,44,45,46,47].

### Limitations

Despite the strengths of the present study, several limitations should be acknowledged. First, the retrospective design and relatively small sample size may limit the generalizability of the findings. Although an a priori power analysis indicated that the sample size was sufficient to detect a large effect size, the limited number of participants should be considered when interpreting the results. Second, condylar position was evaluated using two-dimensional lateral cephalometric radiographs rather than three-dimensional imaging modalities such as CBCT. Consequently, mediolateral condylar displacement and rotational changes could not be assessed, and anatomical superimposition may have influenced landmark identification despite careful standardization procedures. As a result, subtle positional changes may have been underestimated or obscured, and the findings should therefore be interpreted as reflecting changes in the overall condyle–fossa relationship rather than precise three-dimensional condylar displacement. Third, although the magnitude of mandibular setback was recorded, the potential influence of individual surgical movement magnitude on postoperative condylar adaptation could not be fully evaluated because of the limited sample size. Furthermore, no clinical assessment of TMJ symptoms or function, such as pain, joint sounds, or mandibular range of motion, was performed. Therefore, the radiographic findings cannot be directly correlated with postoperative TMJ symptomatology or functional outcomes. Finally, the absence of a control group prevented differentiation between delayed manifestations of surgical repositioning and adaptive changes occurring during the postoperative follow-up period. In addition, because only patients with complete radiographic records at all observation periods (T0, T1, and T2) could be included, a degree of selection bias cannot be entirely excluded.

From a clinical perspective, these findings suggest that manual proximal segment positioning may provide acceptable immediate postoperative condylar stability, but may not fully prevent subsequent changes in the condyle–fossa relationship over time. Therefore, meticulous intraoperative seating of the condyle, careful fixation of the proximal and distal segments, and postoperative monitoring of joint adaptation remain important components of BSSRO treatment. When available, adjunctive techniques that facilitate reproducible condylar positioning may further contribute to minimizing unintended positional changes and improving long-term joint stability.

## 5. Conclusions

Manual positioning of the proximal segment during bilateral sagittal split ramus osteotomy may provide acceptable immediate postoperative condylar stability, but may not completely maintain the preoperative condylar position throughout the postoperative treatment period. Although no significant immediate postoperative changes were observed, significant changes in condylar position within the glenoid fossa became evident at the final follow-up examination. Because none of the Cartesian coordinate measurements demonstrated significant differences, these findings should be interpreted as alterations in the condyle–fossa relationship, with a tendency toward a more anterior position within the glenoid fossa, rather than direct evidence of true condylar displacement. These findings provide clinically relevant insight into condylar positional changes after BSSRO and support the need for further prospective studies using three-dimensional imaging modalities.

## Figures and Tables

**Table 1:** Lateral cephalometric measurements before surgery (T0) and immediately after surgery (T1).

Measurements	T0		T1		*p*
	Min–Max	Mean ± SD	Min–Max	Mean ± SD	
SNA (°)	74–85	79.90 ± 3.15	78–82	79.80 ± 2.67	0.736
SNB (°)	75–89	81.07 ± 3.38	74–84	80.00 ± 2.91	0.081
ANB (°)	−5–1	−1.57 ± 2.24	0–6	2.14 ± 1.79	0.001
Overjet (mm)	−6–1	−2.21 ± 2.01	1–4	2.21 ± 0.70	0.001
Overbite (mm)	−9–5	−0.29 ± 3.73	1–2	1.36 ± 0.50	0.170
GoGN/SN (°)	27–38	34.86 ± 3.74	27–38	33.07 ± 3.22	0.007

Values are presented as minimum–maximum and mean ± standard deviation (SD). T0: preoperative; T1: immediate postoperative. SNA, SNB, ANB, and GoGn/SN are expressed in degrees; overjet and overbite in millimeters. *p* < 0.05.

**Table 2:** Comparison of condylar and joint space measurements at T0, T1, and T2.

Measurement	T0	T1	T2	*p*	Significant Difference
	Mean ± SD	Mean ± SD	Mean ± SD		
CPI	1.07 ± 13.80	1.54 ± 13.89	7.02 ± 11.12	0.006	T2 > T0, T1
AJS (mm)	1.69 ± 0.51	1.71 ± 0.56	1.57 ± 0.46	0.016	T0, T1 > T2
SJS (mm)	1.77 ± 0.39	1.78 ± 0.37	1.76 ± 0.32	0.913	
PJS (mm)	1.68 ± 0.26	1.71 ± 0.29	1.76 ± 0.22	0.117	
Ca-Y (mm)	13.92 ± 3.45	13.98 ± 3.85	14.00 ± 3.91	0.052	
Cs-Y (mm)	16.59 ± 3.62	16.86 ± 4.09	16.89 ± 4.18	0.184	
Cp-Y (mm)	19.33 ± 3.52	19.36 ± 4.06	19.46 ± 4.08	0.245	
Ca-X (mm)	19.39 ± 2.79	20.17 ± 2.52	19.99 ± 3.00	0.223	
Cs-X (mm)	18.39 ± 2.84	19.27 ± 2.52	18.84 ± 2.99	0.157	
Cp-X (mm)	19.69 ± 2.82	20.46 ± 2.62	20.14 ± 2.90	0.424	

Values are presented as mean ± SD. CPI: Condyle Position Index; AJS: anterior joint space; SJS: superior joint space; PJS: posterior joint space. X and Y represent horizontal and vertical distances according to the constructed Cartesian coordinate system. *p* < 0.05.

## Data Availability

The data presented in this study are not publicly available due to ethical and privacy restrictions, as they are derived from anonymized patient radiographic records obtained from institutional archives. Data may be made available from the corresponding author upon reasonable request and with permission of the relevant ethics committee.

## Abbreviations

TMJ	Temporomandibular Joint
MSSP	Muskuloskeletal Stable Position
BSSRO	Bilateral Sagittal Split Ramus Osteotomy
AJS	Anterior Joint Space
SJS	Superior Joint Space
PJS	Posterior Joint Space
CPI	Condyle Position Index
Ca	Anterior Condylar Point
Cp	Posterior Condylar Point
Cs	Most Superior Point of the Condyle
Ca′	Projection Point on the Anterior Wall of the Glenoid Fossa
Cp′	Projection Point on the Posterior Wall of the Glenoid Fossa
ICC	Intraclass Correlation Coefficient
